# Lipidomic analysis of plasma samples from women with polycystic ovary syndrome

**DOI:** 10.1007/s11306-014-0726-y

**Published:** 2014-08-17

**Authors:** Zeina Haoula, Srinivasarao Ravipati, Dov J. Stekel, Catharine A. Ortori, Charlie Hodgman, Clare Daykin, Nick Raine-Fenning, David A. Barrett, William Atiomo

**Affiliations:** 1 Centre for Analytical Bioscience, School of Pharmacy, University of Nottingham, Nottingham, UK; 2 School of Medicine, Queen’s Medical Centre, University of Nottingham, Nottingham, NG7 2UH UK; 3 School of Biosciences, University of Nottingham, Nottingham, LE12 5RD UK; 4 MetaboConsult UK, Derby, UK

**Keywords:** Polycystic ovary syndrome, Lipidomics, Biomarkers, Menstrual cycle

## Abstract

Polycystic ovary syndrome (PCOS) is a common disorder affecting between 5 and 18 % of females of reproductive age and can be diagnosed based on a combination of clinical, ultrasound and biochemical features, none of which on its own is diagnostic. A lipidomic approach using liquid chromatography coupled with accurate mass high-resolution mass-spectrometry (LC-HRMS) was used to investigate if there were any differences in plasma lipidomic profiles in women with PCOS compared with control women at different stages of menstrual cycle. Plasma samples from 40 women with PCOS and 40 controls aged between 18 and 40 years were analysed in combination with multivariate statistical analyses. Multivariate data analysis (LASSO regression and OPLS-DA) of the sample lipidomics datasets showed a weak prediction model for PCOS versus control samples from the follicular and mid-cycle phases of the menstrual cycle, but a stronger model (specificity 85 % and sensitivity 95 %) for PCOS versus the luteal phase menstrual cycle controls. The PCOS vs luteal phase model showed increased levels of plasma triglycerides and sphingomyelins and decreased levels of lysophosphatidylcholines and phosphatidylethanolamines in PCOS women compared with controls. Lipid biomarkers of PCOS were tentatively identified which may be useful in distinguishing PCOS from controls especially when performed during the menstrual cycle luteal phase.

**Electronic supplementary material:**

The online version of this article (doi:10.1007/s11306-014-0726-y) contains supplementary material, which is available to authorized users.

## Introduction

Polycystic ovary syndrome (PCOS) remains an enigma, despite the fact that it affects 5–18 % of females of reproductive age (Dunaif 1997; Legro et al. 1998; March et al. 2010). The heterogenous disorder is characterized by chronic oligo-anovulation, and hyperandrogenaemia, associated with morphologically abnormal ovaries with numerous small follicular cysts (Banaszewska et al. 2006). Most symptoms of PCOS are irregular or non-existent period, very light and very heavy bleeding during period, infertile, over weight (Bogdonov et al. 2008). Although significant progress has been made in our understanding of PCOS, there are still challenges in unravelling its complexity. The diagnosis of PCOS is based on a combination of clinical, ultrasound and biochemical features, none of which on its own is diagnostic (Rotterdam ESHRE/ASRM-Sponsored PCOS Consensus Workshop Group 2004); a single unifying mechanism has yet to be found. New experimental approaches are therefore required to help address some of the scientific and clinical challenges (Pasquali et al. 2011; Atiomo et al. 2009a, b). In PCOS, genomic, proteomic and more recently metabolomic studies (Urbanek et al. 1999; Hughes et al. 2006; Luque-Ramirez and San Millan 2006; Goodarzi 2008; Chen et al. 2011; Matharoo-Ball et al. 2007; Ma et al. 2007; Choi et al. 2007; Corton et al. 2008; Insenser et al. 2010; Atiomo and Daykin 2012; Zhao et al. 2012) have identified new pathways and molecular targets that offer promise in unravelling the complexity of PCOS.

A number of human diseases are linked with abnormal lipid metabolism including obesity, atherosclerosis, diabetes and fatty liver disease (Han et al. 2000; Kim et al. 2010; Reddy and Rao 2006) and it is likely that metabolic dysfunction observed in PCOS will result in the generation of lipid biomarkers of the disease in blood. The emergence of lipidomics as a distinct field (Wenk 2005), coupled with the advancement of new analytic platforms (Quehenberger and Dennis 2011) offer new opportunities to investigate changes in lipid profiles from patients compared to controls so as to determine biologically significant deviations from the norm. The global view of lipid metabolism offered by lipidomics has the potential to improve our understanding of PCOS disease processes (Meikle and Christopher 2011) and could potentially introduce new biomarkers into clinical practice for early diagnosis of disease and improved patient management (Postle 2012).

In a recent metabolomics study, Zhao et al. (2012) found changed levels of carbohydrates, amino acids and lipids in PCOS patients. Specifically, they noted that the levels of long-chain fatty acids, triglycerides and very low-density lipoprotein were elevated, while phosphatidylcholine and HDL concentrations were reduced in PCOS patients as compared with controls. The aim of this study was further investigate these distinctive changes in the plasma lipid profiles between women with PCOS and controls using validated lipidomics methodology.

## Materials and methods

### Reagents and materials

A Milli-Q water purification system (Millipore, MA, USA) was used in the preparation of deionized water (18.2 MΩ). Acetonitrile and chloroform were HPLC grade purchased from Fischer Scientific (Loughborough, UK). Methanol (LC–MS grade) and ammonium acetate were purchased from Sigma-Aldrich (Dorset, UK). Isopropanol (LC–MS grade) and ethanol AR grade were obtained from Fischer Scientific (Loughborough, UK). Lipid chemical standards (including eicosanoids, endocannabinoids and prostaglandins, listed in full in the Supplementary information Table S1) were purchased from Axxora (Bingham, UK) and IDS PLC (Boldon, UK).

### Sample collection

#### Ethics approval and study design

Ethics committee (Institutional review board) approval was obtained for this study from the UK East Midlands Health Research Authority, Research Ethics Committee (REC reference 10/H0408/69). This was a cross-sectional study at the University hospital department of Obstetrics and Gynaecology at the Queen’s Medical Centre Campus, Nottingham University Hospitals NHS Trust, Nottingham, in which 40 women with PCOS and 40 controls aged between 18 and 40 years were prospectively recruited.

#### Recruitment of participants and controls

PCOS was defined as women with two or more of the following in the absence of other endocrine causes of oligo-and/or anovulation (Rotterdam ESHRE/ASRM-Sponsored PCOS Consensus Workshop Group 2004): oligo-and/or anovulation, clinical and/or biochemical signs of hyperandrogenism and polycystic ovary (PCO) morphology on ultrasound scan. Clinical evidence of hyperandrogenism was defined as clinical history of hirsutism and biochemical evidence of hyperandrogenism was defined as a free androgen index of 5 or more. Age matched women without PCOS who had regular 21–35 day menstrual cycles were used as controls. Controls were identified from patients and female members of staff who volunteered to participate. Specific inclusion criteria for controls included the following; regular 21–35 day menstrual cycles, no ultrasound evidence of PCO, no evidence of hyperandrogenism and not currently using hormonal contraception. Women with PCOS were identified from the gynaecology/endocrine and the fertility clinics at the Queen’s Medical Centre, Nottingham. Women with PCOS who met the inclusion criteria were approached verbally or in writing, asking if they would like to participate in this study. A patient information sheet was provided and following informed consent, they were recruited into the study. Women with PCOS or controls with any of the following were excluded from the study: thyroid disease, current pregnancy, delivery or miscarriage occurring within the preceding 3 months, recent surgery (within 3 months), history of myocardial infarction, use of aspirin or heparin, sex steroid therapy, a history of haematological disease, malignancy or liver disease, hyperprolactinaemia, a history of thrombosis and recent viral illness. No body mass index limits were a pre-requisite for inclusion or exclusion. Amongst the women with PCOS, five women had a history of asthma, one had hypertension, one had clinical depression, one had hypothyroidism and one had sleep apnoea. Amongst the controls, two women had a history of asthma, two had uterine fibroids, one had mitral valve prolapse, one eczema, one psoriasis, one clinical depression and one trigeminal neuralgia. With respect to medicament ingest, amongst the women with PCOS, four were using asthma inhalers, one was on ramipril, one was on thyroxin, one was on amitriptyline and two were on folic acid. Amongst the controls, two women were on asthma inhalers, one on gabapentin and one was on Prozac. With respect to diet, all women were fasted before blood samples were collected. We did not collect any data on whether or not women were vegetarian or not.

#### Interventions

Women were invited into the hospital where the following interventions occurred. A clinical interview was undertaken to elicit the reproductive, medical, drug, family and social histories. Anthropometric measurements for height, weight, body mass index (BMI), waist and hip circumference were obtained. Fasting blood samples were then taken for endocrine (testosterone, sex hormone binding globulin, luteinising hormone, follicle stimulating hormone, thyroid function tests, 17-hydroxyprogesterone, prolactin, insulin, glucose, cholesterol, triglycerides and high density lipoproteins) and lipidomic assays and a pelvic ultrasound was performed to measure the endometrial thickness, ovarian follicle count, size and blood flow. Women with PCOS were invited once in for the fasting blood tests and ultrasound scans. PCOS samples were taken once on any day of the menstrual cycles because of the menstrual irregularity associated with PCOS. Control women without PCOS were however invited three times in their menstrual cycles (follicular, mid cycle and secretory phase) to enable an evaluation of cycle variability in the lipid profiles. Fasting blood samples were collected in pre-chilled lithium heparin tubes and centrifuged within 30 min at 2,000×*g* and 4 °C for 10 min. Plasma was separated and immediately stored at −80 °C until analysis.

### Sample preparation

Lipids were extracted from plasma samples (50 µL) by adding 0.5 mL of cold (−20 °C) chloroform/methanol (1:2), the frozen plasma sample being allowed to thaw in the presence of the extraction solvent. After brief vortex-mixing (20 s), 0.5 mL of water was added and the tube contents mixed again for 10 min, centrifuged at 1,000×*g* for 10 min at 4 °C. An aliquot of the lower lipophilic phase (100 µL) was removed and mixed with an equal volume of isopropanol prior to analysis. A pooled QC sample was prepared by mixing 20 µL aliquots taken from each individual plasma study sample and treated exactly as described for the study samples.

### Liquid chromatography-mass spectrometry lipidomic analysis

#### Chromatography

Chromatographic separations were performed on an ACE 3 C18 HPLC column (150 × 2.1 mm, 3 µm particle size; Aberdeen, UK) maintained at a temperature of 40 °C and a flow rate of 300 µL/min. Mobile phases consisted of (A) 60:40 acetonitrile:10 mM aqueous ammonium acetate and (B) 90:10 isopropanol:10 mM ammonium acetate in acetonitrile. A binary gradient from 30 to 97 % B was used with a total run time of 15 min. The injection volume was 10 µL.

#### Mass spectrometry

Mass spectrometry was performed on an Orbitrap Exactive MS (Thermo Fisher Scientific, USA) acquiring data simultaneously in full scan ion mode (*m*/*z* 100–1200, resolution 25,000, AGC 1e^6^) in both positive and negative modes. The capillary voltage was maintained at 25 V in the positive ion mode and at 27 V in the negative ion mode. All other interface settings used were same for both positive and negative modes. The voltages of tube lens and skimmer in positive mode were set to 115 and 22 V respectively. Negative mode voltages of tube lens and skimmer were set to 140 and 28 V respectively. The flow rates of sheath gas, auxillary gas and sweep gas for both positive negative modes were adjusted to 30, 15 and 5 (arbitrary units). The capillary temperature and heater temperature were maintained at 350 and 300 °C respectively in both positive and negative modes.

### Data analysis and metabolite identification

Raw LC–MS data from the PCOS and control group samples were acquired using Xcalibur v2.1 software (Thermo Scientific, Hemel Hemstead UK), the control groups including samples from each of the three phases of the menstrual cycle (follicular, mid-cycle and secretory). The raw data for each sample analysis consisted of 5–8,000 resolvable LC–MS signals identified by *m*/*z*, retention time and ion signal intensity. The full datasets from PCOS group and the control groups were imported and pre-processed by Progenesis CoMet v.1.2 software (Nonlinear Dynamics, Newcastle upon Tyne, UK). The performance of the analytical method was validated by monitoring a representative set of plasma lipids in pooled quality control (QC) samples for retention time-shifts, relative standard deviations (RSD%) of peak areas and mass accuracy. Multivariate data analysis was used to investigate changes in the plasma-lipid profiles between the PCOS and individual menstrual cycle datasets using principal component analysis (PCA), orthogonal partial least squares discriminant analysis (OPLS-DA) using SIMCA-P v12 (Umetrics, Umea, Sweden) and Logistic Lasso regression from the R package glmnet (Friedman et al. 2010). Initial models based on the entire datasets (n = 40) were cross validated (leave one out method) and further prediction models were based on randomly selected training (n = 20) and test sets (n = 20) with sensitivity and specificity calculations reported. Any biomarkers associated with BMI were then excluded from the final model to eliminate the potential confounding effects of obesity. Tentative identification of key lipid biomarkers was achieved by using accurate mass determinations within a narrow *m*/*z* range (1–5 mDa) to search appropriate metabolite databases: lipid maps (http://www.lipidmaps.org/) and the Human Metabolome database (http://www.hmdb.ca/).

## Results

### Demographic data

Table 1 shows the demographic and endocrine features of the 40 selected women with PCOS compared with 40 controls. Samples taken in the follicular phase from controls were analysed for endocrine data. There was no significant difference in the ages of both groups. However women with PCOS had a significantly higher BMI, LH, testosterone and free androgen index and lower FSH levels as consistent with the diagnosis of PCOS.

**Table 1:** Demographic and endocrine data comparing PCOS to controls

Variable mean (±SD)	PCOS N = 40	Controls N = 40	*p* value	q value
Age (years)	29.2 (4.5)	30.6 (5.0)	0.26	0.36
BMI (kg/m^2^)	29.5 (6.1)	25.4 (5.9)	7.4 × 10^−4^	3.2 × 10^−3^*
LH (IU/L)	13.8 (11.6)	5.20 (2.40)	3.9 × 10^−7^	6.7 × 10^−6^*
FSH(IU/L)	5.69 (2.87)	7.50 (3.73)	1.4 × 10^−3^	4.9 × 10^−3^*
Testosterone (mmol/L)	2.18 (0.76)	1.47 (0.54)	2.5 × 10^−5^	2.1 × 10^−4^*
SHBG (nmol/L)	52.8 (29.9)	61.6 (26.7)	0.068	0.17
Free androgen index	5.03 (2.91)	2.56 (1.77)	5.8 × 10^−5^	3.3 × 10^−4^*
PRL (pmol)	225 (122)	193 (78)	0.33	0.41
TSH (mlU/L)	1.85 (1.06)	2.37 (1.33)	0.024	0.069
FT4 (pmol/L)	15.5 (3.0)	15.1 (1.9)	0.83	0.83
17-OHP (nmol/L)	6.76 (5.65)	4.84 (2.75)	0.28	0.36
Fasting insulin (μU/mL)	7.18 (6.21)	5.15 (5.54)	0.092	0.20
Fasting glucose (mmol/L)	4.54 (0.42)	4.66 (0.44)	0.22	0.36
Insulin/glucose ratio	1.40 (1.18)	1.22 (1.22)	0.25	0.36
Cholesterol (mmol/L)	4.49 (0.78)	4.84 (0.82)	0.49	0.52
TG (mmol/L)	1.00 (0.57)	0.835 (0.297)	0.20	0.36
HDL (mmol/L)	1.51 (0.31)	1.58 (0.31)	0.38	0.43

*SD* standard deviation, *BMI* body mass index, *SHBG* sex hormone binding globulin, *FAI* free androgen index, *LH* luteinising hormone, *FSH* follicle stimulating hormone, *PRL* prolactin, *TSH* thyroid stimulating hormone, *FT4* free thyroxine, *17-OHP* 17 hydroxy progesterone, *TG* triglycerides, *HDL* high density lipoproteins

*p* values have been calculated using the non-parametric Mann–Whitney test as many measures are not normally distributed. q value corrections ^22^ are included to correct for multiple testing and are more indicative of significance; measures with significant differences (q value <0.05) have been marked with a *

### Validation of LC–MS lipidomic method performance

**Fig. 1:**
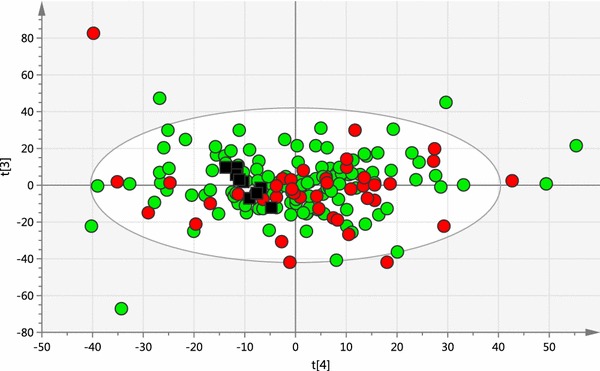
Overview PCA scores plot obtained from all PCOS samples (*red circles*, n = 40), all control samples (*green circles*, n = 120) and all pooled QC samples (*black squares*, n = 9). (R2X = 0.654, Q2 = 0.285, A = 24, total N = 169) (Color figure online)

**Fig. 2:**
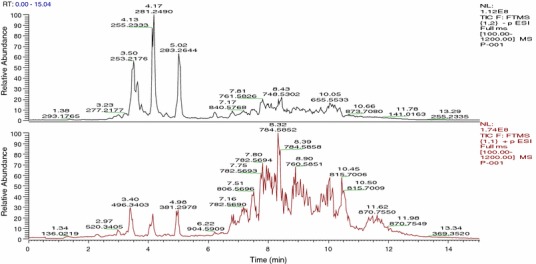
The total ion LC-HRMS chromatogram of a typical PCOS plasma sample extracted with chloroform/methanol in negative (*upper*) and positive (*lower*) ionisation modes. All the chromatograms were recorded in the range of *m*/*z* 100–1,200

The analytical performance of the LC–MS lipidomics method was evaluated using QC samples pooled from aliquots of each study sample. All sample and QC extracts were analysed in a single LC–MS run with pooled QC samples being interspaced with study samples. The pooled QC samples were tightly clustered by PCA analysis (Fig. 1). In the QC datasets the % RSD values of peak areas of selected typical plasma lipids were in the range of 7.6–13.2 %, retention time shifts were less than 0.07 min, the mass accuracy deviation was less than 1 mDa in positive ion mode and less than 2 mDa in negative ion mode (Supplementary Information Table S2). These results validate the LC–MS lipidomics analytical performance during the analysis of the study samples. Typical LC–MS chromatograms obtained from plasma extracts are shown in Fig. 2.

### Plasma lipidomics analysis of PCOS and menstrual cycle control samples

**Fig. 3:**
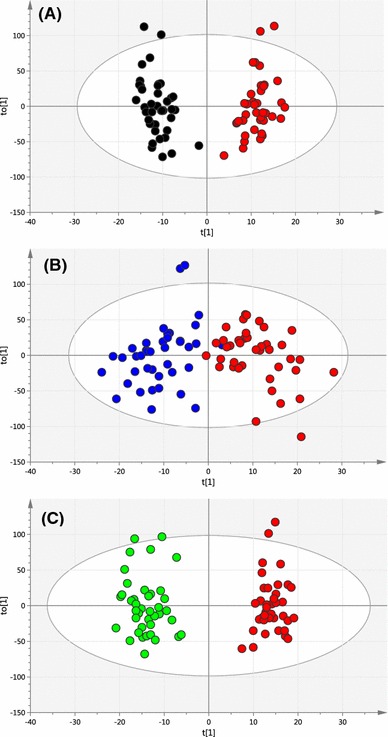
OPLS-DA scores plot obtained between all PCOS samples (*red circles*, n = 40) and **a** control follicular-cycle samples (*black circles*, n = 40) (R2X = 0.389, R2Y = 0.939, Q2 = 0.259, A = 1 + 4 + 0); **b** Control mid-cycle samples (*blue circles*, n = 40) (R2X = 0.314, R2Y = 0.759, Q2 = 0.225, A = 1 + 2 + 0) and **c** control luteal-cycle samples (*green circles*, n = 40). (R2X = 0.417, R2Y = 0.953, Q2 = 0.512, A = 1 + 4 + 0) (Color figure online)

**Fig. 4:**
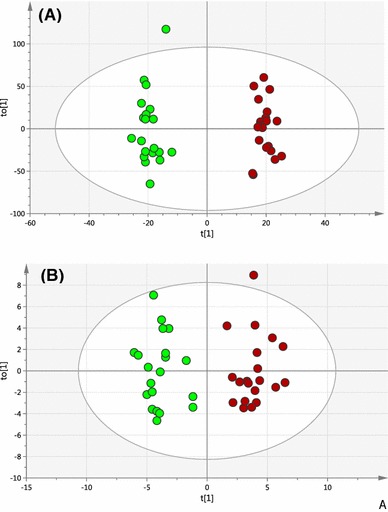
**a** OPLS-DA scores plot (test set) obtained from a random selection of 50 % PCOS (*red circles*) and control luteal-cycle (*green circles*) samples. Model was built using a training set constructed from the complementary control and PCOS sample data. (R2X = 0.367, R2Y = 0.979, Q2 = 0.569, A = 1 + 3 + 0, N = 40). **b** OPLS-DA scores plot obtained based on potential biomarkers only from PCOS samples (*red circles*) and control luteal-cycle samples (*green circles*) (R2X = 0.634, R2Y = 0.903, Q2 = 0.739, A = 1 + 3 + 0, N = 40) (Color figure online)

**Fig. 5:**
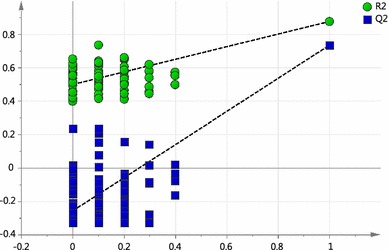
Validation plot obtained from permutation test (n = 100) for the OPLS-DA model of PCOS versus luteal phase. R2 is the explained variance, and Q2 is the predictive ability of the model. The Q2-intercept value was less than 0.05 shows that the model is statistically sound and high predictability of the model is not because of over-fitting data

**Fig. 6:**
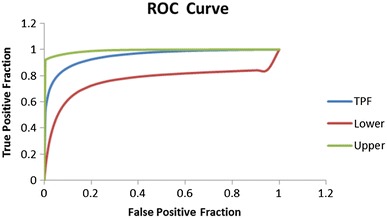
ROC curve is defined as true positive fraction versus false positive fraction. To affirm the validity of prediction OPLS-DA model of PCOS vs luteal phase, area under receiver operating characteristic (ROC) curve was calculated. The area under the curve was 0.95 (an ideal model would have an AUC of 1) which clearly states that the prediction model was robust. *TPF* true false positive, *upper* upper 95 % confidence interval values and *lower* lower 95 % confidence interval values

Complete LC–MS lipidomics datasets were obtained for the PCOS samples (n = 40) and the control samples at the follicular (n = 40), mid-cycle (n = 40) and luteal (n = 40) phases of the menstrual cycle. No differences in plasma lipid profiles could be distinguished between the three control samples sets from different stages of the menstrual cycle using multivariate data analysis. Using supervised multivariate data analysis methods of Lasso regression analysis and OPLS-DA, it was possible to build cross-validated models based on small differences in lipid profiles which could predict between individuals with PCOS and control samples at each stage of the menstrual cycle using the lipidomics datasets (OPLS-DA scores plot, Fig. 3). These models were evaluated by monitoring the goodness of model (R2X) and predictive ability (Q2) values. OPLS-DA model comparing between PCOS and luteal cycle gave better R2X and Q2 values (0.417 and 0.512 respectively) than PCOS vs follicular-cycle control groups (R2X = 0.389, Q2 = 0.259) or PCOS vs mid-cycle control groups (R2X = 0.314, Q2 = 0.225). Where certain PCOS biomarkers were associated with BMI (as was the case with PCOS vs luteal phase controls) these were excluded from the models. However, when a more stringent model validation process was applied involving randomly selected training (n = 20) and test sets (n = 20) only with the PCOS vs luteal phase samples was a valid disease model confirmed (Fig. 4, OPLS-DA example). The results of multivariate data analysis and the specificity and sensitivity of the models generated are shown in Table 2. Overall, the two multivariate data analysis methods, OPLS-DA and LASSO regression, gave similar results. In addition, a permutation test (n = 100) was conducted to evaluate the prediction model (Fig. 5) and the Q2-intercept value (−0.27) from the prediction model at less than 0.05 shows that the good predictive ability of the model was not because of over-fitting of the model to the complex data sets. Moreover, the model was validated by calculating area under receiver operating characteristic (ROC) curve (Eng 2007). The value of area under curve (AUC) was 0.95 which gives added confidence of the model (Fig. 6).

**Table 2:** Prediction of PCOS status based on OPLS-DA and LASSO regression models

Analysis method	Datasets used	Cross-validated model		Training/test model	
		Sensitivity (%)	Specificity (%)	Sensitivity (%)	Specificity (%)
Lasso regression	PCOS vs follicular	80	72.5	70	50
	PCOS vs mid-cycle	62.5	60	55	40
	PCOS vs luteal	97.5	100	85	95
OPLS-DA	PCOS vs follicular	92.5	90	55	75
	PCOS vs mid-cycle	60	65	45	65
	PCOS vs luteal	100	100	85	95

### Plasma lipid biomarkers of PCOS

The greatest differences in the plasma lipid profiles were observed between the PCOS and luteal menstrual cycle control groups in both Lasso regression and OPLS-DA models. Potential lipid biomarkers were therefore derived from the mass spectrometry *m*/*z* variables which had the most significant contribution to both of these models (Table 3). These *m*/*z* variables, which are derived from the high resolution mass spectrometry data obtained from the lipidomics analysis, are represented as an accurate mass of an individual lipid (generally to within 1–2 mDa) and have undergone deconvolution to remove adducts, isotopes and other confounding factors resulting from mass spectrometry detection. The exact mass of the biomarkers coupled with derived empirical molecular formula were then used to interrogate appropriate metabolic databases and to provide tentative identification of the lipid species. The top 25 lipid species are shown in Table 3 together with a structural code from Lipid Maps (http://www.lipidmaps.org/) or the Human Metabolome database (http://www.hmdb.ca/).

**Table 3:** Biomarkers showing differences between PCOS patients and luteal phase control subjects

Biomarker MW (Da)	Formula	Mass difference (mDa)	Lipid tentative identification	OPLS-DA analysis	Lasso regression analysis	PCOS change ↓↑
286.2297	C_20_H_30_O	0.61	Unknown	✓	✓	↑
351.2443	C_17_H_37_NO_4_S	4.70	Unknown	✓	✓	↑
716.4992	C_39_H_73_O_9_P	0.50	Unknown	✓	✓	↑
829.6561	C_47_H_92_NO_8_P	1.70	PE (42:1)	✓	✓	↓
725.4995	C_40_H_72_NO_8_P	1.50	PC (32:4)	✓	✓	↓
691.5516	C_38_H_78_NO_7_P	1.20	Plasmalogen (30:0)	✓	✓	↓
757.2191	C_33_H_41_O_20_	2.90	Unknown	✓	✓	↑
815.5829	C_48_H_82_NO_7_P	1.90	Plasmalogen (40:7)	✓	✓	↑
620.5379	C_39_H_72_O_5_	0.002	DG (36:2)	✓	✓	↑
608.538	C_38_H_72_O_5_	1.70	Unknown	✓	✓	↑
936.8146	C_61_H_108_O_6_	0.28	TG (58:5)	✓		↑
910.7989	C_59_H_106_O_6_	0.73	TG (56:4)	✓		↑
754.5989	C_43_H_83_N_2_O_6_P	1.14	SM (d18:0/20:2)	✓		↑
876.7206	C_57_H_96_O_6_	1.90	TG (54:7)	✓		↑
868.752	C_56_H_100_O_6_	0.68	TG (52:4)	✓		↑
820.7519	C_53_H_104_O_5_	1.68	TG (50:0)	✓		↑
618.5223	C_39_H_70_O_5_	0.25	DG (36:3)	✓		↑
732.6145	C_41_H_85_N_2_O_6_P	1.30	SM (d18:0/18:0)	✓		↑
705.5308	C_38_H_76_NO_8_P	0.86	PC (30:0)	✓		↓
719.5465	C_39_H_78_NO_8_P	1.01	PE (34:0)	✓		↓
495.3324	C_24_H_50_NO_7_P	0.67	LysoPC (16:0)	✓		↓
519.3324	C_26_H_50_NO_7_P	0.24	LysoPC (18:2)	✓		↓
521.3481	C_26_H_52_NO_7_P	0.58	LysoPC (18:1)	✓		↓
523.3637	C_26_H_54_NO_7_P	0.07	LysoPC (18:0)	✓		↓

Mass difference (mDa) is the difference in exact monoisotopic mass between the measured value and the theoretical value

*PC* phosphatidylcholine, *DG* diacylglycerol, *TG* triglyceride, *SM* sphingomyelin, *PE* phosphatidylethanolamine, *LysoPC* lysophosphatidylcholine

**↑↓** increase or decrease of biomarker in PCOS group compared with luteal control group

Plasma samples of PCOS patients were distinguished from the luteal menstrual cycle control plasma by a combination of small changes in lipid composition. Lipid biomarkers which were consistently increased or decreased in women with PCOS included increases in triglycerides (TG) and sphingomyelins (SM) and decreases in lysophosphatidylcholines (LysoPC) and phosphatidylethanolamines (PE) (Table 3). Some lipids in the model (PC phosphatidylcholine; DG diacylglycerol) had both increased and decreased fragment ions of the same lipid families (Table 3).

## Discussion

As previously observed by Zhao et al. (2012) we did not find a single lipid biomarker in plasma for PCOS, but rather there was a pattern of change in the plasma lipid profiles which distinguished the control group from the PCOS patients. The observed changes in our study were relatively small, requiring detailed multivariate data-analysis to separate the PCOS group from the controls. The strengths of our study are the rigorous validation methods used in the data analysis to minimise the risks of false positives and the use of validated LC-HRMS for ensured a high sensitivity and specificity. Plasma samples of PCOS patients showed significantly increased levels of mainly membrane lipids including triglycerides and sphingomyelins, and decreased levels of lysophosphatidylcholines and phosphatidylethanolamines when PCOS samples were compared with control samples taken in the luteal phase of the menstrual cycle which is consistent with the changes in lipids observed by Zhao et al. (2012).

It is currently not clear why the differences identified in the study were much more prominent in comparisons between PCOS and controls samples taken in the luteal phase of the menstrual cycle, it may however be a reflection of biochemical changes which occur following ovulation in the luteal phase of the menstrual cycle in most women with regular menstrual cycles in contrast to anovulatory women with PCOS. We could not identify any previously published studies investigating the variation of sphingomyelins lysophosphatidylcholines and phosphatidylethanolamines in the menstrual cycle, but there had been previous studies on triglycerides, in which the data appeared conflicting. In one study (Punnonen 1978), of ten healthy women with regular menstrual cycles tested for levels of total serum cholesterol, triglycerides, phospholipids, and estradiol three times during one menstrual cycle (during menstruation, at ovulation, and in the luteal phase), lipid variations during the menstrual cycle were minimal and serum cholesterol, triglycerides, and phospholipids did not correlate with changes in the serum estradiol levels. On the other hand, two studies were identified showing lower triglyceride levels in the luteal phase of the menstrual cycle compared to the mid-follicular phase (Mumford et al. 2010) or menses (Woods and Graham 1986). Another possible explanation for these results includes the possibilities of type 1 or type 2 statistical errors, and only a repeat study on larger cohorts of patients would clarify this.

The pattern of changes in some of the lipid profiles observed may however not be inconsistent with the PCOS phenotype. The finding of increased plasma triglyceride levels for example was consistent with previously published studies (Zhao et al. 2012; Wild et al. 2011) on plasma lipid changes in PCOS. Sphingomyelin is found in animal cell membranes, especially in the membranous myelin sheath that surrounds some nerve cell axons, and is thought to have a role in signal transduction (Kolesnick 1994). In a recently published study of metabolomic profiling of plasma from women with PCOS compared with controls of a similar body mass index using proton NMR metabolomics, a signal was identified in the NMR spectra that was thought to be possibly consistent with a higher plasma level of sphingomyelin (Sun et al. 2012). Lysophosphatidylcholine is a minor phospholipid in the cell membrane but is present in significant amounts in the blood plasma (Munder et al. 1979). Reduced lysophosphatidylcholines have as far as we know not been previously found in women with PCOS. Lysophosphatidylcholines (LPC) (Munder et al. 1979) are however derived from partial hydrolysis of phosphatidylcholines which have been shown to be reduced in women with PCOS compared with controls (Zhao et al. 2012; Sun et al. 2012).

In our study BMI was higher in PCOS compared with control patients which could be perceived as non-ideal, however there is debate about the use of BMI matching in PCOS studies. For example Bloom et al. (2007)argued that matching did not offer advantages over independent control selection with regard to study validity (i.e., confounding bias) and our data partially supports this in that there was no obvious variation identified when the lipid profiles taken from control women in the three different phases of the menstrual cycle were compared with each other. This provided further justification the random timing of blood tests in the PCOS group in addition to the fact that irregular menstrual cycles in women with PCOS would have made it impossible to perform similar repetitive blood tests. In addition, other surrogate markers of the obesity including insulin, glucose, insulin/glucose ratios, HDL cholesterol and triglycerides were not significantly different in women with PCOS compared with controls in our study. This was further supported by the results of Lasso regression analysis which showed that the predictive models were independent of BMI.

The clinical significance of this study is that we have identified a panel of potential lipid biomarkers of PCOS which may be useful in distinguishing them from controls especially when performed during the menstrual cycle luteal phase. It is hoped that the publication of this study will stimulate interest in this area by independent research groups and encourage further validation of lipid metabolites and pathways altered in PCOS.

## Electronic supplementary material

Below is the link to the electronic supplementary material.

Supplementary material 1 (DOCX 23 kb)
