# Supplementary material for: Lipidomic analysis of plasma samples from women with polycystic ovary syndrome
Source: Metabolomics. 2014 Aug 17;11(3):657–66. doi: 10.1007/s11306-014-0726-y (PMC4419155; doi:10.1007/s11306-014-0726-y)
Supplement: Supplementary file 1 — Supplementary material 1 (DOCX 23 kb) [file 11306_2014_726_MOESM1_ESM.docx]

**SUPPLEMENTARY INFORMATION**

**Lipidomic analysis of plasma samples from women with polycystic ovary syndrome**

**Zeina Haoula ^2^, *Srinivasarao Ravipati ^1^, Dov J. Stekel ^3^, Catharine A. Ortori ^1^, Charlie Hodgman ^3^, Clare Daykin* *^1^*^,^ *^4^, Nick Raine-Fenning ^2^, David A. Barrett ^1^ and William Atiomo ^2^*

* Joint first authors

**Institutional Affiliations**

^1^Centre for Analytical Bioscience, School of Pharmacy, University of Nottingham, UK

^2^School of Medicine, University of Nottingham, UK

^3^School of Biosciences, University of Nottingham, LE12 5RD, UK

^4^MetaboConsult UK, Derby, UK

**Address for Correspondence**

William Atiomo, School of Medicine, Queen's Medical Centre, Nottingham, NG7 2UH, UK

Email: william.atiomo@nottingham.ac.uk

Tel: 0115 82 30712

Fax: 0115 82 30704

**Supplementary** **Table S1:** List of endocannabinoids, eicosanoids and prostaglandins

| **Compound Name** | **Molecular Formula** |
| --- | --- |
| **Endocannabinoids** |  |
| Arachidonyl ethanolamide (*N*-(2-hydroxyethyl)-5Z,8Z,11Z,14Z-eicosatetraenamide, anandamide, AEA) | C_22_H_37_NO_2_ |
| 2-arachidonyl glycerol (2-AG, (5Z,8Z,11Z,14Z)-5,8,11,14-eicosatetraenoic acid, 2-hydroxy-1-(hydroxymethyl)ethyl ester) | C_23_H_38_O_4_ |
| Palmitoyl ethanolamide (PEA, *N*-(2-hydroxyethyl)-hexadecanamide) | C_18_H_37_NO_2_ |
| Oleoyl ethanolamide (OEA, *N*-(2-hydroxyethyl)-9Z-octadecenamide) | C_20_H_39_NO_2_ |
| **Eicosanoids** |  |
| 5,6-DHET ((±)5,​6-​dihydroxy-​8Z,​11Z,​14Z-​eicosatrienoic acid) | C_20_H_34_O_4_ |
| 8,9-DHET ((±)8,​9-​dihydroxy-​5Z,​11Z,​14Z-​eicosatrienoic acid) |  |
| 11,12-DHET ((±)11,​12-​dihydroxy-​5Z,​8Z,​14Z-​eicosatrienoic acid) |  |
| 14,15-DHET ((±)14,​15-​dihydroxy-​5Z,​8Z,​11Z-​eicosatrienoic acid) |  |
| 12-HPETE (12-​hydroperoxy-​5Z,​8Z,​10E,​14Z-​eicosatetraenoic acid) | C_20_H_32_O_4_ |
| 5-HETE ((±)5-​hydroxy-​6E,​8Z,​11Z,​14Z-​eicosatetraenoic acid) | C_20_H_32_O_3_ |
| 8-HETE ((±)8-​hydroxy-​5Z,​9E,​11Z,​14Z-​eicosatetraenoic acid) |  |
| 9-HETE ((±)-​9-​hydroxy-​5Z,​7E,​11Z,​14Z-​eicosatetraenoic acid) |  |
| 11-HETE ((±)11-​hydroxy-​5Z,​8Z,​12E,​14Z-​eicosatetraenoic acid) |  |
| 12-HETE ((±)12-​hydroxy-​5Z,​8Z,​10E,​14Z-​eicosatetraenoic acid) |  |
| 15-HETE ((±)15-​hydroxy-​5Z,​8Z,​11Z,​13E-​eicosatetraenoic acid) |  |
| 16-HETE (16S-​hydroxy-​5Z,​8Z,​11Z,​14Z-​eicosatetraenoic acid) |  |
| 19-HETE (19R-​hydroxy-​5Z,​8Z,​11Z,​14Z-​eicosatetraenoic acid) |  |
| 20-HETE (20-​hydroxy-​5Z,​8Z,​11Z,​14Z-​eicosatetraenoic acid) |  |
| 5,6-EET ((±)5(6)-​epoxy-​8Z,​11Z,​14Z-​eicosatrienoic acid) | C_20_H_32_O_3_ |
| 8,9-EET ((±)8(9)-​epoxy-​5Z,​11Z,​14Z-​eicosatrienoic acid) |  |
| 11,12-EET ((±)11(12)-​epoxy-​5Z,​8Z,​14Z-​eicosatrienoic acid) |  |
| 14,15-EET ((±)14(15)-​epoxy-​5Z,​8Z,​11Z-​eicosatrienoic acid) |  |
| 5,6-EET-EA (N-​(2-​hydroxyethyl)-​(±)5(6)-​epoxy-​8Z,​11Z,​14Z-​eicosatrienamide) | C_22_H_37_NO_3_ |
| 14,15-EET-G (2-​hydroxy-​1-​(hydroxymethyl)ethyl ester-​13-​(3-​pentyloxiranyl)-​5Z,​8Z,​11Z-​tridecatrienoic acid) | C_23_H_38_O_5_ |
| Arachidonic acid (5Z,​8Z,​11Z,​14Z-​eicosatetraenoic acid) | C_20_H_32_O_2_ |
| **Prostaglandins** |  |
| PGD2-EA (N-​(2-​hydroxyethyl)-​11-​oxo-​9α,​15S-​dihydroxy-​prosta-​5Z,​13E-​dien-​1-​amide) | C_22_H_37_NO_5_ |
| PGE1-EA (N-​(2-​hydroxyethyl)-​9-​oxo-​11α,​15S-​dihydroxy-​prost-​13E-​en-​1-​amide) | C_22_H_39_NO_5_ |
| PGE2-EA (N-​(2-​hydroxyethyl)-​9-​oxo-​11α,​15S-​dihydroxy-​prosta-​5Z,​13E-​dien-​1-​amide) | C_22_H_37_NO_5_ |
| PGF2α-EA (N-​(2-​hydroxyethyl)-​9α,​11α,​15S-​trihydroxy-​prosta-​5Z,​13E-​dien-​1-​amide) | C_22_H_39_NO_5_ |

Supplementary Table S2: Analysis of pooled QC samples for PCOS biomarker study. Precision in peak areas and retention times and mass deviation was recorded for individual lipids present. Samples were analysed on ACE 3 C18 HPLC column (150×2.1 mm, 3µm particle size) with total run time of 15 min

| **Lipid** | **%RSD peak area** | **%RSD retention time** | **Mass deviation mDa** |
| --- | --- | --- | --- |
| HETEs | 10.03 | 0.57 | 1.25 |
| PGF2α | 8.55 | 0.53 | 0.88 |
| LysoPC(20:4) | 9.66 | 0.31 | 0.25 |
| LysoPC(18:2) | 11.86 | 0.79 | 0.31 |
| α or γ linolenic acid | 12.32 | 0.42 | 1.05 |
| Docohexaenoic acid | 9.72 | 0.29 | 0.98 |
| LysoPC(15:0) | 12.69 | 0.45 | 0.66 |
| LysoPC(16:0) | 14.22 | 0.38 | 0.72 |
| Arachidonic acid | 13.13 | 0.32 | 0.55 |
| LysoPC(18:1) | 13.01 | 0.36 | 0.63 |
| Palmitoleic acid | 7.01 | 0.28 | 1.23 |
| PEA | 14.23 | 0.53 | 0.79 |
| Linoleic acid | 9.61 | 0.36 | 0.66 |
| LysoPC(18:0) | 11.14 | 0.30 | 0.12 |
| LysoPE(22:6) | 10.10 | 0.30 | 0.45 |
| DHETs | 6.02 | 0.30 | 1.43 |
| Palmitic acid | 10.51 | 0.20 | 1.12 |
| Palmitoyl glycerol | 12.13 | 0.29 | 0.91 |
| Oleic acid | 10.23 | 0.24 | 0.16 |
| Heptadecanoic acid | 8.67 | 0.19 | 0.72 |
| 13,14 Dihydro PGF1α | 13.84 | 0.30 | 0.81 |
| Stearic acid | 9.48 | 0.26 | 0.36 |
| Eicosanoic acid | 8.74 | 0.20 | 0.52 |

PC phosphatidylcholine; PE phosphatidylethanolamine; LysoPC lysophosphatidylcholine; HETE hydroxy ​eicosatetraenoic acid; PGF2α prostaglandin F2α; PEA palmitoyl ethanolamide; LysoPE lysophosphatidylethanolamine; DHET dihydroxy ​eicosatrienoic acid; PGF1α prostaglandin F1α;
